# Impact of sweep gas flow on extracorporeal CO_2_ removal (ECCO_2_R)

**DOI:** 10.1186/s40635-019-0244-3

**Published:** 2019-03-25

**Authors:** Stephan Strassmann, Michaela Merten, Simone Schäfer, Jonas de Moll, Daniel Brodie, Anders Larsson, Wolfram Windisch, Christian Karagiannidis

**Affiliations:** 1 Department of Pneumology and Critical Care Medicine, Cologne-Merheim Hospital, ARDS and ECMO Centre, Kliniken der Stadt Köln gGmbH, Witten/Herdecke University Hospital, Ostmerheimer Strasse 200, D-51109 Cologne, Germany; 2 Division of Pulmonary, Allergy and Critical Care, Columbia University College of Physicians and Surgeons/New York-Presbyterian Hospital, New York, NY USA; 3 Hedenstierna Laboratory, Anesthesiology and Intensive Care, Department of Surgical Sciences, Uppsala University, Uppsala, Sweden

**Keywords:** ARDS, Extracorporeal carbon dioxide removal, COPD, ECCO_2_R, ECMO

## Abstract

**Background:**

Veno-venous extracorporeal carbon dioxide (CO_2_) removal (vv-ECCO_2_R) is increasingly being used in the setting of acute respiratory failure. Blood flow rates range in clinical practice from 200 mL/min to more than 1500 mL/min, and sweep gas flow rates range from less than 1 to more than 10 L/min. The present porcine model study was aimed at determining the impact of varying sweep gas flow rates on CO_2_ removal under different blood flow conditions and membrane lung surface areas.

**Methods:**

Two different membrane lungs, with surface areas of 0.4 and 0.8m^2^, were used in nine pigs with experimentally-induced hypercapnia. During each experiment, the blood flow was increased stepwise from 300 to 900 mL/min, with further increases up to 1800 mL/min with the larger membrane lung in steps of 300 mL/min. Sweep gas was titrated under each condition from 2 to 8 L/min in steps of 2 L/min. Extracorporeal CO_2_ elimination was normalized to a PaCO_2_ of 45 mmHg before the membrane lung.

**Results:**

Reversal of hypercapnia was only feasible when blood flow rates above 900 mL/min were used with a membrane lung surface area of at least 0.8m^2^. The membrane lung with a surface of 0.4m^2^ allowed a maximum normalized CO_2_ elimination rate of 41 ± 6 mL/min with 8 L/min sweep gas flow and 900 mL blood flow/min. The increase in sweep gas flow from 2 to 8 L/min increased normalized CO_2_ elimination from 35 ± 5 to 41 ± 6 with 900 mL blood flow/min, whereas with lower blood flow rates, any increase was less effective, levelling out at 4 L sweep gas flow/min. The membrane lung with a surface area of 0.8m^2^ allowed a maximum normalized CO_2_ elimination rate of 101 ± 12 mL/min with increasing influence of sweep gas flow. The delta of normalized CO_2_ elimination increased from 4 ± 2 to 26 ± 7 mL/min with blood flow rates being increased from 300 to 1800 mL/min, respectively.

**Conclusions:**

The influence of sweep gas flow on the CO_2_ removal capacity of ECCO_2_R systems depends predominantly on blood flow rate and membrane lung surface area. In this model, considerable CO_2_ removal occurred only with the larger membrane lung surface of 0.8m^2^ and when blood flow rates of ≥ 900 mL/min were used.

## Background

Veno-venous extracorporeal CO_2_ removal (vv-ECCO_2_R) is increasingly being used in the setting of acute hypercapnic and hypoxemic respiratory failure to facilitate protective ventilation, allowing early extubation or even to avoid invasive mechanical ventilation [1–9]. In this context, ECCO_2_R blood flow rates range from 200 mL/min to more than 1500 mL/min [10–12], depending on the primary treatment aim [2]. Much effort has been spent in the last decade to increase CO_2_ removal capacity by modifying the circuit and the system [13–15]. With respect to possible technical modifications, blood flow rates, sweep gas flow rates and membrane lung surface areas may be manipulated by the treating team at the bedside.

Among these, blood flow rates were reported to have the strongest impact on CO_2_ removal capacity [16, 17]. It was recently demonstrated that applying blood flow rates of 1000 mL/min with an appropriate membrane lung surface can remove about 50% of total CO_2_ production and, therefore, can correct even severe respiratory acidosis [18]. Less is known about the influence of sweep gas flow in the setting of typical ECCO_2_R blood flow rates, especially in regard to different membrane lung surface areas. This is of particular importance since membrane lung surfaces from 0.4–1.3 m^2^ also clearly impact on CO_2_ removal capacities independent of the blood flow level [18].

Furthermore, CO_2_ removal capacity is not only dependent on technical circumstances of the system, but also on the absolute CO_2_ content of the blood. Most of the CO_2_ of the body is stored as bicarbonate (HCO_3_^−^) in slow reacting compartments such as bone [19, 20] and is, therefore, not directly accessible for CO_2_ removal. Only 1–5% of the total CO_2_ content is dissolved in the blood and can thus be extracorporeally removed. Importantly, the amount of soluble CO_2_ is strongly correlated to the absolute CO_2_ level in venous and arterial blood. Therefore, normalization of CO_2_ removal to a PCO_2_ of 45 mmHg before the membrane lung allows for the comparison of different settings independent of the amount of PCO_2_ before the membrane lung [21].

The aim of the current study was, therefore, to determine the effect of varying degrees of sweep gas flow on normalized CO_2_ removal using a clinically typical range of blood flows for ECCO_2_R via a porcine model of severe hypercapnic respiratory failure.

## Material and methods

### Veno-venous-extracorporeal CO_2_ removal (ECCO_2_R) techniques

For the vv-ECCO_2_R system, two different membrane lungs (Getinge, Maquet Cardiopulmonary Care, Rastatt, Germany) based on the Rotaflow® platform were used. The membrane lungs consisted of a polymethylpentene membrane with surface areas of 0.4 and 0.8 m^2^, respectively. These surface areas have been chosen due to the previous results [18] and current clinical practice in centres applying ECCO_2_R with different systems (0.4 and 0.8 m^2^). Both membrane lungs have a comparable rhomboid design. The systems were primed with normal saline solution. Heparin (5000 IE) was added to the running system, and bolus application of 5000 IE every 2–3 h was used during the running of the systems to avoid clotting.

For venous access, a 23 Fr Bicaval Avalon ELITE Dual Lumen Cannula® (Getinge Group, Maquet Cardiopulmonary Care, Rastatt, Germany) was inserted into the right jugular vein. Correct placement of the cannula was confirmed by echocardiography. The membrane lung with 0.4 m^2^ was measured with system blood flow rates of 300, 600 and 900 mL/min, whereas the membrane lung with 0.8 m^2^ was measured with system blood flow rates from 300 to 1800 mL/min in increasing steps of 300 mL/min, respectively. For each single membrane lung surface area and blood flow rate, sweep gas flow was titrated in steps of 2 L/min increasing from 2 L/min to 8 L/min with a delivered oxygen fraction of 1.0.

### Animal model

The study was approved by the Animal Research Committee of Uppsala University/Sweden (ethical approval number C77/16). Pigs (body weight = 39 ± 2 kg) were anaesthetized with IV ketamine 25–50 mg/kg/h, midazolam 90–80 μg/kg/h, fentanyl 3–6 μg/kg/h, and rocuronium 2.5–5.0 mg/kg/h was added when adequate anaesthesia was ascertained by lack of response of painful stimulation between the front hooves. The trachea was intubated with a cuffed endotracheal tube (inner diameter, 7 mm). The pigs were ventilated with a Servo-i ventilator (Maquet Critical Care, Solna, Sweden). Body temperature was kept at 38 °C throughout the study period by use of a heater cooling unit (Maquet Critical Care, Solna, Sweden). Arterial blood was sampled from the left carotid artery. The estimated CO_2_ production in this setting is about 200–280 mL/min in pigs [22, 23], which is comparable to a resting adult human.

### Study design

Vv-ECCO_2_R was performed in nine pigs following endotracheal intubation, mechanical ventilation and induction of hypercapnia by increased dead space ventilation, as described previously [16, 18]. Anatomical dead space was increased by adding an additional tube between the endotracheal tube and the “Y” piece of the ventilator circuit. The length of the additional tube was titrated until hypercapnia was induced with a target PaCO_2_ value ranging between 90 and 110 mmHg. The animals were ventilated in a volume-controlled mode with a tidal volume of 220–250 mL, a positive end-expiratory pressure of 5 cmH_2_O and a breathing frequency of 14–16/min. The dead space fraction and minute ventilation were subsequently maintained for the entire duration of the experimental period after having reached equilibrium with the target PaCO_2_ of 90 to 110 mmHg.

The experiments were performed in each pig in a standardized fashion. PaCO_2_ was equilibrated between every system for at least 30 min. When PaCO_2_ was stable at the target, blood flow was increased stepwise from 300 mL/min to 1800 mL/min. At each blood flow level, sweep gas flow was titrated from 2 to 8 L/min in steps of 2 L/min.

### CO_2_ and blood gas measurement

Blood gas analysis was performed with an ABL 800, Radiometer, (Copenhagen, Denmark) with separate measurement for haemoglobin. Extracorporeal CO_2_ removal was calculated as reported in detail in previous studies [18, 24, 25]. Of note, to compare the amount of CO_2_ removal at different pre-membrane PCO_2_ levels, the extracorporeal CO_2_ removal was normalized to a PCO_2_ of 45 mmHg. This normalization allowed comparison of CO_2_ removal rates at different PCO_2_ levels, since CO_2_ removal in general is markedly dependent on the amount of soluble CO_2_ (and thus of PCO_2_) in the blood [26].

In more detail, the authors are aware that it is difficult to normalize CO_2_ removal to a specific PaCO_2_ precisely, since CO_2_ can also be dissolved from bicarbonate. However, the carbon dioxide dissociation curve is quite linear in the PCO_2_-interval in our study, i.e. when PCO_2_ is reduced (in our study by the ECCO_2_R system), this will reduce the blood content of carbon dioxide according to the slope of the curve. Another factor that affects the CO_2_ removal is the Haldane effect; since blood PO_2_ increases in the ECCO_2_R-membrane, the Haldane effect will cause a parallel shift downwards of the CO_2_ dissociation curve, and thus the difference between two PCO_2_ levels will be more pronounced [27–29].

### Statistics

For statistical analysis, GraphPad Prism 7 for Macintosh computer (La Jolla, CA 92037, USA) was used. Data were tested for normality using the Kolmogorov-Smirnov test. All data are given as mean with standard deviation.

## Results

**Fig. 1:**
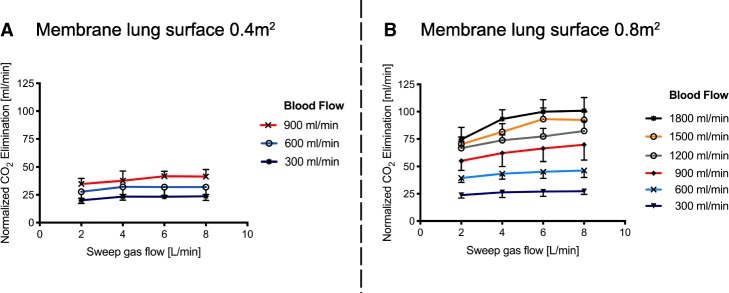
Normalized extracorporeal elimination of carbon dioxide (CO_2_) depending on blood flow and sweep gas flow with a membrane lung surface of 0.4m^2^ (**a**) and 0.8m^2^ (**b**). Normalized CO_2_ elimination was calculated by normalizing the partial pressure of carbon dioxide before the membrane lung to 45 mmHg. The normalized extracorporeal CO_2_ elimination was plotted against sweep gas flow. Blood flow was titrated from 300 to 900 mL/min (**a**) and 300 to 1800 mL/min (**b**). Each data point represents the mean and standard deviation among nine pigs

**Fig. 2:**
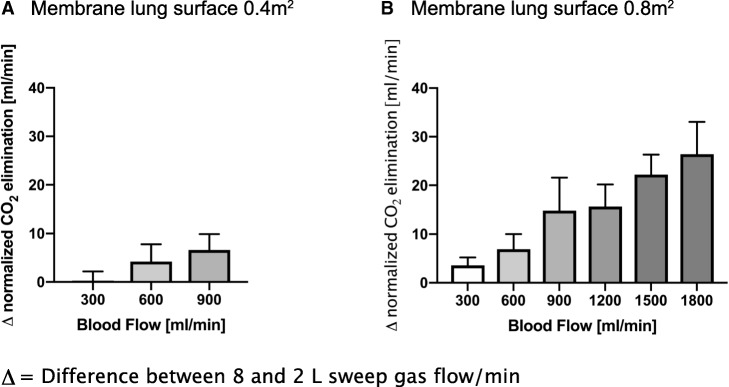
Difference in normalized extracorporeal elimination of carbon dioxide (CO_2_) between 8 and 2 L sweep gas flow/min depending on blood flow and membrane lung surface (0.4m^2^ (**a**) and 0.8m^2^ (**b**))

**Fig. 3:**
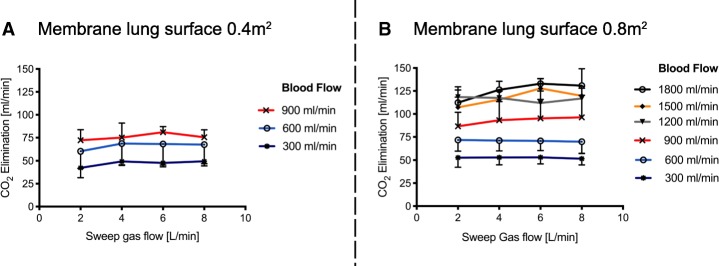
Extracorporeal elimination of carbon dioxide (CO_2_) depending on blood flow and sweep gas flow with a membrane lung surface of 0.4m^2^ (**a**) and 0.8m^2^ (**b**). The extracorporeal CO_2_ elimination was plotted against sweep gas flow. Blood flow was titrated from 300 to 900 mL/min (**a**) and 300 to 1800 mL/min (**b**). Each data point represents the mean and standard deviation among nine pigs. Of note, compared to Fig. 1, the values shown are the absolute and non-normalized CO_2_ values, demonstrating the physiological variance of the CO_2_ amount before the membrane lung in an animal ECCO_2_R experiment

**Fig. 4:**
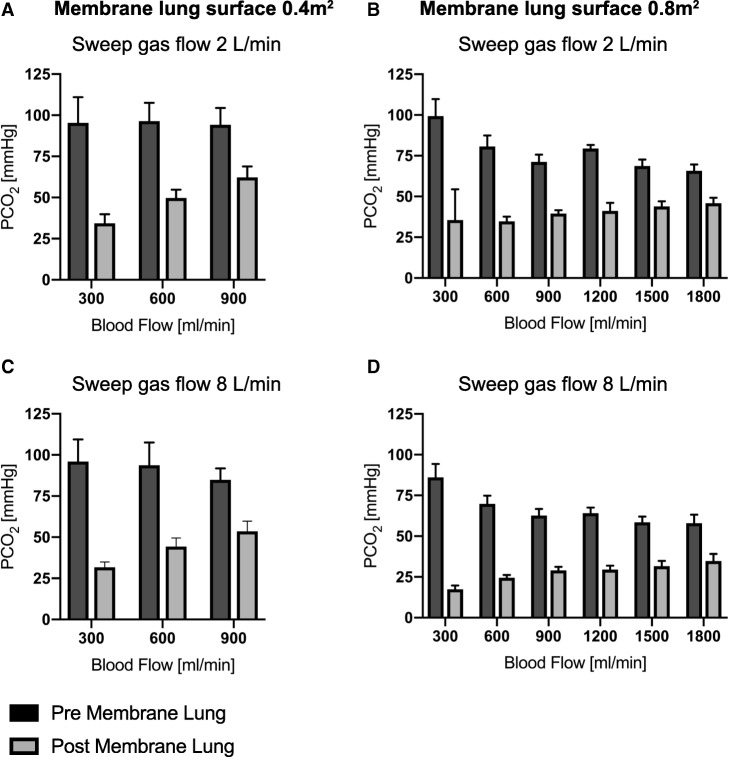
PCO_2_ pre- and post-membrane lung under different blood flow conditions (300–1800 mL/min) with two different membrane lung surface areas (0.4m^2^ (**a** and **c**) and 0.8m^2^ (**b** and **d**)). Each data point represents the mean and standard deviation among nine pigs

For independent comparison, normalized CO_2_ elimination (Figs. 1 and 2 and Table 1) was calculated by normalizing the partial pressure of CO_2_ before the membrane lung to 45 mmHg to compensate for different PCO_2_ levels pre-membrane lung as previously described [18, 21]. Non-normalized extracorporeal CO_2_ removal (Fig. 3) with different CO_2_ levels pre-membrane lung are presented in Fig. 4, demonstrating a blood flow-, sweep gas flow- and membrane lung-dependent CO_2_ removal capacity.

**Table 1:** Normalized extracorporeal CO_2_ elimination (absolute values are given in millilitre per minute). Normalized CO_2_ elimination was calculated by normalizing the partial pressure of carbon dioxide before the membrane lung to 45 mmHg. Values are given as mean with standard deviation

	Sweep gas flow 2 L/min	Sweep gas flow 4 L/min	Sweep gas flow 6 L/min	Sweep gas flow 8 L/min
ML surface 0.4m^2^				
BF 300 mL/min	20.1 ± 2.9	23.3 ± 3.2	23.2 ± 2.3	23.7 ± 3.7
BF 600 mL/min	27.8 ± 5.8	32.2 ± 6.9	32.0 ± 7.6	32.0 ± 6.6
BF 900 mL/min	34.8 ± 4.9	37.7 ± 8.7	41.7 ± 4.5	41.4 ± 6.3
ML surface 0.8m^2^				
BF 300 mL/min	23.8 ± 2.8	26.3 ± 4.7	27.0 ± 4.3	27.4 ± 3.0
BF 600 mL/min	39.4 ± 4.1	43.4 ± 5.0	45.2 ± 6.1	46.3 ± 6.4
BF 900 mL/min	54.9 ± 8.5	62.1 ± 12.1	66.3 ± 11.9	69.8 ± 14.1
BF 1200 mL/min	66.6 ± 6.4	73.8 ± 4.9	77.4 ± 7.2	82.3 ± 9.0
BF 1500 mL/min	70.2 ± 6.6	81.5 ± 7.5	93.2 ± 10.2	92.4 ± 7.6
BF 1800 mL/min	74.5 ± 11.0	93.3 ± 8.4	100.0 ± 11.0	100.9 ± 12.0

The membrane lung with a surface of 0.4 m^2^ allowed a maximum normalized CO_2_ elimination rate of 41 ± 6 mL/min with 8 L/min sweep gas flow and 900 mL blood flow/min (Fig. 1 and Table 1). Increase of a sweep gas flow from 2 to 8 L/min, at its maximal effect, increased the normalized CO_2_ elimination from 35 ± 5 to 41 ± 6 mL/min at 900 mL blood flow/min, whereas at lower blood flow rates, any increase was less effective, levelling out early on at 4 L sweep gas flow/min.

The membrane lung with a surface area of 0.8m^2^ showed comparable results within blood flow rates from 300 to 600 mL/min, although the absolute amount of CO_2_ removal was higher than with a surface area of 0.4 m^2^ (Fig. 1 and Table 1). However, the effect of increased sweep gas flow from 2 to 8 L/min was comparable to the smaller membrane lung. When progressing from 900 mL/min up to 1800 mL/min, there was an increasing effect of increasing sweep gas flow on extracorporeal CO_2_ elimination (Table 1). Blood flow rates of 900 mL/min showed an increase in normalized CO_2_ elimination from 55 ± 9 to 70 ± 14 mL/min (delta normalized CO_2_ elimination of 15 mL/min) by increasing the sweep gas flow from 2 to 8 L/min. With each step of increase in blood flow (steps of 300 mL/min), the influence of increased sweep gas flow also increased. Accordingly, the maximum delta between 2 and 8 L sweep gas flow/min (26 ± 7 mL/min) occurred with 1800 mL blood flow/min.

Of note, Fig. 4 demonstrates a linearly decreasing CO_2_ pre-membrane lung with increasing blood flow rates (Figs. 4a and b) with a more pronounced effect with higher sweep gas flow rates (Figs. 4c and d).

## Discussion

The present study provides the most comprehensive overview to date of the influence of sweep gas flow on the CO_2_ removal capacity of ECCO_2_R systems with varying blood flow rates and membrane lung surface areas as used in this model. It adds to previous animal studies on technical determinants of successful vv-ECCO_2_R. While two former studies using a comparable study design have primarily focused on the impact of the blood flow rate [16] and the membrane lung surface area [18], respectively, on the capability of the system to remove CO_2_, the present study aimed to determine the role of the sweep gas flow in the intersection of these three key components of vv-ECCO_2_R. The main finding is that the impact of the sweep gas flow varies depending on the size of the membrane lung and the chosen blood flow rate. Thus, the impact of the sweep gas flow rate is clinically negligible when using a small membrane lung surface area of 0.4 m^2^ in addition to blood flow rates of up to 900 mL/min.

In contrast, the impact of the sweep gas flow rate appears to be clinically more critical when using a larger membrane lung surface of 0.8 m^2^. In this case, the difference of the CO_2_ elimination, following mathematical normalization to 45 mmHg when comparing 8 and 2 L sweep gas flow per minute, is at least 10 mL/min or even higher at a blood flow rate of 900 mL/min, and this difference appears to gradually increase when using even higher blood flow rates. Eventually, this difference reached approximately 25 mL/min at a blood flow rate of 1800 mL/min, taking into account that normalized CO_2_ elimination is calculated, thus the real CO_2_ removal capacity might be even higher if CO_2_ before the membrane lung is above 45 mmHg.

The present findings confirm one of the previous porcine studies on pathophysiological and technical considerations of ECCO_2_R [16]. This study had already signified that the capability of extracorporeal CO_2_ removal rises with increasing sweep gas flow rates. However, the experiments of increasing sweep gas flow rates in this former study were performed with a fixed blood flow rate of 1000 mL/min. Therefore, the current study adds to the existing knowledge in so far as the increased capability to extra-corporally remove CO_2_ at higher sweep gas flow rates is substantially greater with higher blood flow rates, but negligible with a blood flow below 600 mL/min.

However, in view of the current and the previous findings, the amount of blood flow still appears to play the most important role for the success of vv-ECCO_2_R. Nevertheless, current evidence, including the present trial, now suggests that there is a complex interplay between the three major selectable regulating variables, i.e. blood flow rate, sweep gas flow rate and the membrane lung surface area.

The present study has some important clinical implications: First, severe hypercapnia is most sufficiently handled by ECCO_2_R with a large enough membrane lung surface area (≥ 0.8 m^2^) using higher blood flow as well as sweep gas flow rates. Second, to achieve the desired results for vv-ECCO_2_R, the main technical determinants of vv-ECCO_2_R (membrane lung surface area, blood flow rate and sweep gas flow rate) should be assessed before starting up the procedure. In addition, in order to adequately compare different methods, these technical conditions have to be determined in advance and reported in clinical studies. This may be most relevant in future clinical trials with adaptive designs which are dependent on the CO_2_ removal capacity.

The present study has some limitations related to the porcine model used and the calculated CO_2_ removal. These limitations have been extensively discussed in the previous publications on vv-ECCO_2_R using a very similar setting for the animal experiments and are, therefore, with reference to the previous studies only briefly addressed [16, 18]. First, data acquired in pigs cannot automatically be transferred into the clinical scenario; however, it has been shown that CO_2_ production in pigs is comparable to CO_2_ production observed in adult humans requiring mechanical ventilation [23], likely allowing comparison. Second, the typical clinical scenario of exacerbated chronic obstructive pulmonary disease (COPD) with severe airflow limitation was not simulated. Therefore, the interaction between vv-ECCO_2_R and mechanical ventilation could not be investigated. Third, the animals were not critically ill and had a normal CO_2_ production in contrast to patients with an acute exacerbation of COPD or with sepsis, who usually have higher CO_2_ production. Therefore, the conclusions regarding the clinical effectiveness of different systems used for vv-ECCO_2_R in the clinical setting must be tempered.

## Conclusions

The influence of sweep gas flow on the CO_2_ removal capacity of ECCO_2_R systems depends predominantly on blood flow rate and membrane lung surface area. In this model, considerable CO_2_ removal occurred only with the larger membrane lung surface of 0.8m^2^ and when blood flow rates of ≥ 900 mL/min were used. Furthermore, it can be emphasized that sweep gas flow has a significant impact on CO_2_ removal capacity in ECCO_2_R, but only if blood flow rates of 900 mL/min and above are applied. Finally, in regard to future ECCO_2_R clinical trials, especially those with adaptive designs, it will be important to understand the influence of sweep gas flow in the intersection of blood flow, membrane lung surface area and sweep gas flow.

## Abbreviations

ML: Membrane lung
vv-ECCO_2_R: Veno-venous extracorporeal CO_2_ removal
